# Water temperature and precipitation stimulate small-sized Japanese eels to climb a low-height vertical weir

**DOI:** 10.1371/journal.pone.0279617

**Published:** 2022-12-27

**Authors:** Manabu Kume, Yudai Yoshikawa, Tomoichiro Tanaka, Shun Watanabe, Hiromichi Mitamura, Yoh Yamashita

**Affiliations:** 1 Field Science Education and Research Center, Kyoto University, Kitashirakawa-Oiwake, Sakyo, Kyoto, Japan; 2 Graduate School of Agriculture, Kyoto University, Kitashirakawa-Oiwake, Sakyo, Kyoto, Japan; 3 Tanaka-Sanjiro Co, Ltd., Ogori, Fukuoka, Japan; 4 Faculty of Agriculture, Kindai University, Nara, Japan; University of Hyogo, JAPAN

## Abstract

Although river-crossing structures can have a detrimental effect on the movement and distribution of anguillid eels (genus *Anguilla*), they have inhabited locations upstream of river-crossing structures in many rivers. However, the growth stage in which the eels start to climb river-crossing structures remains unclear. In this study, we directly observed, using infrared video camera systems, that the Japanese eel (*Anguilla japonica*) climbs a low-height vertical weir as a migratory barrier, confirming the ability of eels to climb over a low-height weir within a river. All eels climbed the weir at night, between sunset and sunrise. We observed that the total length of Japanese eels that climbed the weir ranged from 60 to 140 mm, suggesting that eels inhabiting the upstream area of a weir climbed it when they were small and then lived and grew upstream. Moreover, the general additive model showed considerable effects of water temperature and precipitation on eel climbing, suggesting that water temperature and precipitation are important for its activation. The results of this study also show the effectiveness of infrared video cameras in monitoring eel climbing, which could be applied to monitor fish migratory behavior through fish passages. The findings of this study are useful for the comprehensive management and conservation of wild eel stocks.

## Introduction

River-crossing structures (dams and weirs) have been built on rivers worldwide [1,2]. These structures have serious consequences for adjacent aquatic ecosystems; for instance, they can create physical barriers to the upstream/downstream movement of fish. This has resulted in habitat reduction and fragmentation of freshwater fishes, including diadromous fishes; thus, fish abundance and diversity have decreased, and fish extinction risk has increased [3,4]. Recently, various types of rehabilitation/restoration acts of degraded ecosystems, such as fish ladder installation and dam removal, have been conducted globally to recover longitudinal connectivity throughout watersheds [5,6].

The Japanese eel, *Anguilla japonica*, also faces such threats. This species is a facultative catadromous fish that spawns in open seas and grows in continental waters [7]. Glass eels enter fresh/brackish waters [8], and elvers disperse in waters with wide ranges of salinity to take advantage of potentially suitable habitats [9]. They then grow as yellow eels and spend several years in these waters [10]. Approximately 78.6% of the effective habitats of Japanese eels have been lost in East Asian rivers [11]. One of the reasons for this is the river-crossing structures, which negatively affect eel movement and distribution even if they are low-rise structures [12–15]. Our previous studies have shown that the upstream migration of small-sized eels (total length, i.e., TL < 200 mm) was negatively influenced by the number of low-height weirs, whereas that of larger-sized eels (TL > 200 mm) was negatively influenced by the maximum weir height [13,14]. Moreover, the cumulative heights of weirs higher than 40 cm and 60 cm have been reported to hinder the upstream migration of Japanese eels by less than and more than 240 mm TL, respectively [15]. However, another study reported that dams shorter than 3.0 m in height did not prevent the upstream migration of the American eel *A*. *rostrata* [16]. These studies used a model-based approach to determine the relationships between eel density and dam/weir characteristics (i.e., weir number and/or height) along a river at various scales (reach and unit scales). In these studies, eels were collected upstream of low-height weirs, and although they were less in number compared with those collected downstream of weirs, the data suggested that eels climbed weirs at a certain period during their growth stage. Therefore, it is necessary to examine the exact effects of river-crossing structures on the upstream migration of eels. To undertake effective restoration efforts for the conservation of Japanese eels, we investigated these effects more efficiently by determining the exact stage at which eels start to climb weirs.

Recently, several eel upstream passage solutions have been developed [17]. For example, previous studies have tested a variety of fishways or fish ladders as potential eel upstream passages [18,19]. Additionally, dam/weir removal can aid in better eel upstream movement [20]. Whether such approaches for helping eel upstream migration should be adopted depends on the balance between costs (i.e., time and money) and benefits (i.e., improvement of eel growth and survival and the efficiency of reproductive stock reinforcement). However, river-crossing structures are essential for human well-being and safety (i.e., flood control). Furthermore, there are a huge number of river-crossing structures in Japanese rivers [21]. In small rivers of Japan, several low-height weirs have been constructed to raise water levels for agricultural water use or to stabilize riverbeds, and the mean density of such structures, being larger than 40 cm in height, is 1.6 weirs per km [22]. However, low-height weirs in small rivers generally lack fish passage.

This study used video recording equipment to capture the exact stage in which eels climb weirs and to determine their size, the timing of the climbing, and the effects of environmental conditions on this behavior. Based on a previous study, which suggested that smaller-sized eels were more capable of climbing slopes than larger-sized eels [19], we hypothesized that small-sized yellow Japanese eels climb weirs, whereas large-sized eels do not. Additionally, we explored the environmental factors that stimulate eel climbing. We hypothesized that eels climb weirs at night when water temperature exceeds 13°C. This is because anguillid eels, including Japanese eels, are nocturnal [23,24], and the activity of Japanese eels decreases below 13°C [23].

## Materials and methods

### Ethics statement

Wakayama Prefecture (#04010003–48) permitted setting two infrared video camera systems under articles 24 and 26 in the River Act.

### Study site

**Fig 1:**
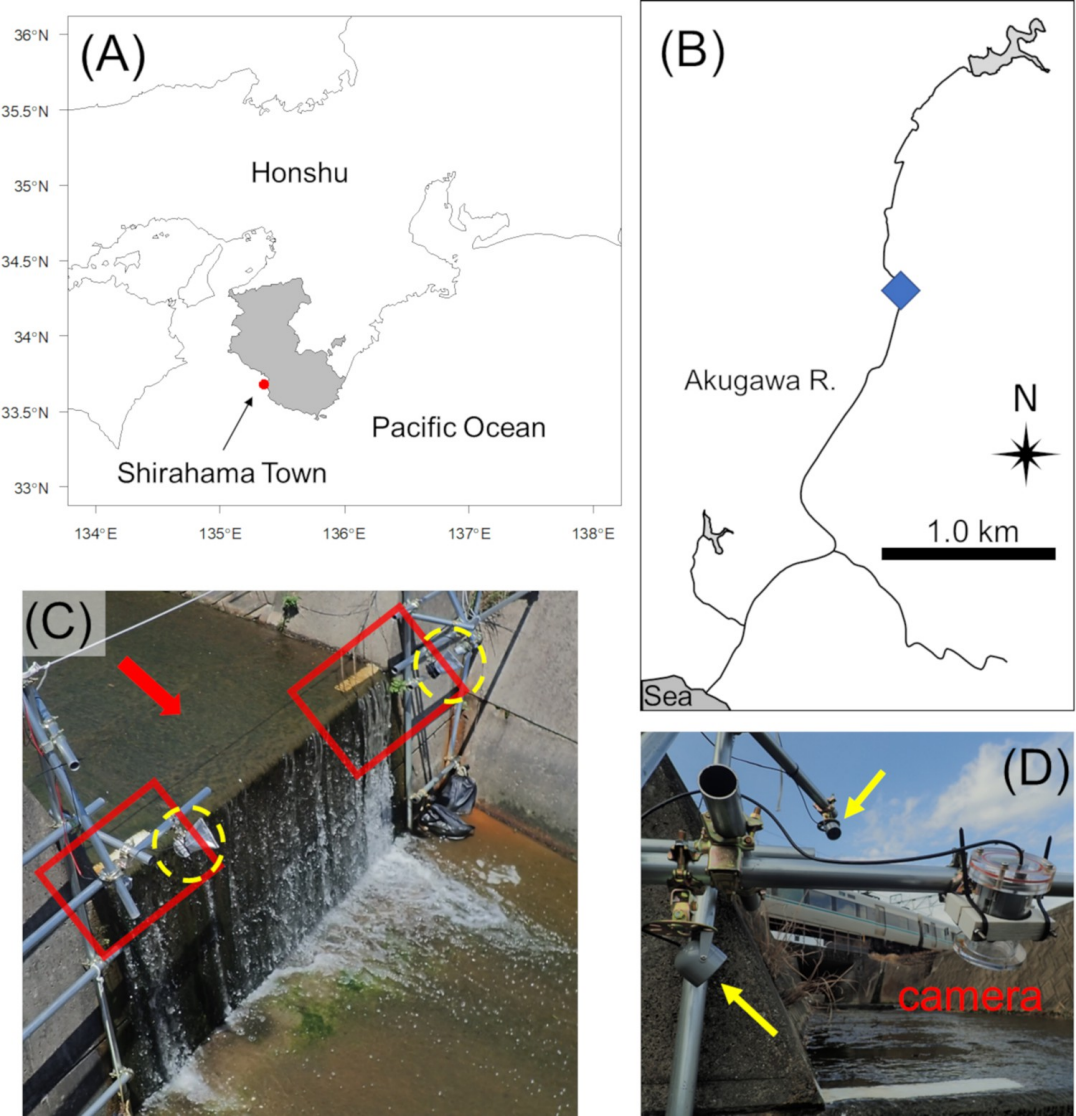
Maps and photographs of the study area. (A) Map showing central Honshu, Japan. The red circle indicates the Akugawa River at Shirahama Town, Wakayama Prefecture. (B) Map of the Akugawa River. The blue square indicates the weir where infrared video camera systems were installed. (C) Photograph of the weir (165 cm height) located at the middle stream of the Akugawa River. The area in the red square indicates the recording angle of the video recorded. Yellow circles with dashed lines indicate infrared video camera systems. The red arrow indicates the flow direction. The white line on the top of the weir was drawn to estimate the TL of eels from the video. (D) Zoom photograph of the infrared video camera system. Yellow arrows indicate infrared lights. Maps were modified using data set (https://geoshape.ex.nii.ac.jp/river/) under CC BY 4.0. Photographs were taken by M.K.

Field surveys were conducted in the Akugawa River (~4.3 km long), Wakayama Prefecture, Japan (Fig 1). The lower course of this river (ca. 2.5 km upstream of the river mouth) runs through a lowland mainly consisting of residential areas. The middle and upper courses of this river (2.5–4.3 km from the river mouth) mainly consisted of farmlands and residential areas. In the study area, there was only one vertical concrete weir; it had a height of 165 cm and was located approximately 2.7 km from the river mouth (Fig 1B and 1C). The wall surface of the weir was eroded and crusted with periphyton mats (~2 mm thickness). The area from the river mouth to the weir had concrete banks on both sides and was intruded with saline water, whereas the areas upstream of the weir were entirely freshwater areas as the weir prevented the saline water intrusion. Our previous study showed that a high density of eels (0.67–1.05 eels/m^2^) with various body lengths (93–641 mm TL) is located in the upper reaches of the Akugawa River [25]. The riverbank of the study area located upstream of the weir mainly consisted of either a masonry revetment on one side and a natural bank on the other side or natural banks on both sides. Areas directly above and below the weir consisted of concrete floors with concrete banks on both sides.

### Video survey

To observe the climbing behavior of eels, we set two infrared video camera systems (CSSTC ver.2.21, Tanaka Sanjiro Co., Ltd., Fukuoka, Japan) on the left and right sides of the weir (Fig 1C). Both cameras were installed at a height of 70 cm from the top of the weir and 50 cm downstream from the weir, and at an angle of approximately 45° from the horizontal. The angle of view was approximately 50 cm × 100 cm, and both the upper part and vertical wall of the weir were monitored. We recorded continuously for 24 hours every day from February 13 to May 31, 2021 at a resolution of 1280 × 720 pixels and 30 fps. As anguillid eels are nocturnal [23,24], two infrared lights per camera were lit at night to observe the climbing behavior of eels. To estimate the TL of eels from the video, we painted a white line (AquaCort, HERMETIC, Tokyo, Japan) on top of the weir (Fig 1C and 1D).

In this study, we recorded videos for 5,520 hours. Due to equipment failure, the camera on the left and right sides of the weir did not record from February 15 to 17 and February 13 to 14, respectively. From the video, we counted the number of eels that successfully climbed the weir, confirmed that they could reach the top of a weir, and recorded the date and time of the successful climbing by each eel. It took approximately 200 hours to check the video played at 32x speed. Additionally, the TL of the eels was approximated to the nearest 10 mm using ImageJ ver.1.53k.

### Collection of environmental data

Water temperature below the weir was measured every 30 minutes using a data logger (UA-002-64, Onset Computer Corp., Bourne, MA, USA) from February 14 to May 31, 2021. Hourly precipitation data (mm/h) at the “Nanki-Shirahama” observation point (33°39ʹ43ʺN, 135°21ʹ47ʺE), the nearest observation point to the Akugawa River, were downloaded from the database of the Japan Meteorological Agency [26]. Daily moon illumination values, as an index of the moon phase, were obtained using the lunar illumination function in the R “lunar” package [27] and ranged from 0 (new moon) to 1 (full moon).

### Statistical analyses

To examine the factors affecting the eel upstream migration timing, we used a general additive model (GAM) with a Tweedie distribution (the *gam* function in the *mgcv* package R package [28]) because of the multiple zeros in the dataset. In this analysis, we used the dataset from February 14 to May 31, in which 24-hour videos were recorded. The number of eels that successfully climbed the weir was the dependent variable, and the water temperature, precipitation (mm/d), and lunar illumination were the predictor variables (S1 Data). We used the daily average water temperatures for the analysis because of the time lag between the time it rains and the time the river discharge changes. Furthermore, we used 24-hour precipitation data from 0, 12, and 24 h before precipitation (i.e. precipitation_*t*_, precipitation_*t-12h*_, and precipitation_*t-24h*_, respectively); precipitation_*t*_ is 24-hour precipitation from 0:00–24:00 on day *t*; precipitation_*t-12h*_ is 24 hour-precipitation from 12:00 at one day before day *t* to 12:00 on day *t* and precipitation_*t-24h*_ is 24 hour-precipitation from 0:00–24:00 one day before day *t*. All analyses used the statistical significance level of 0.05. All statistical tests were performed using R software version 4.1.0 [29].

## Results

### Environmental variables

**Fig 2:**
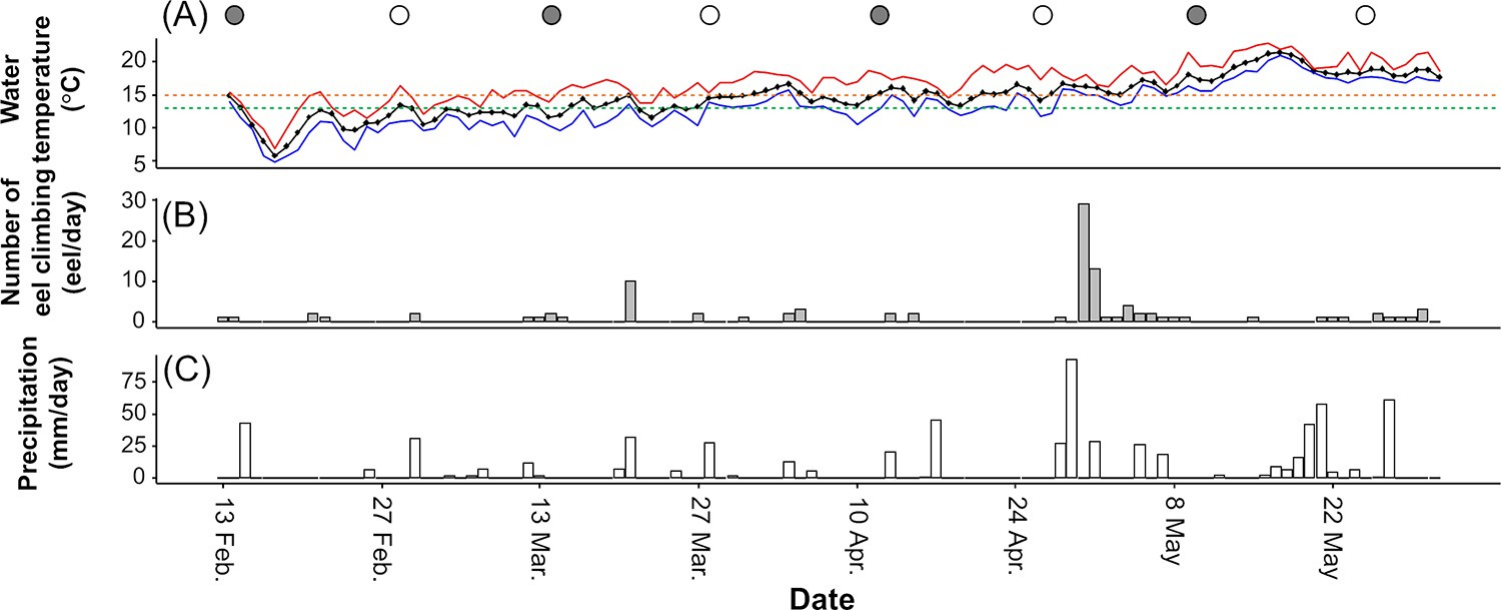
Temporal changes in the environmental conditions and the number of eels that successfully climbed the weir. (A) average water temperature (black plots and line) with minimum (blue line) and maximum (red line) temperatures. Green and orange dashed lines indicate 13°C—the threshold at which eel behavioral activity decreases [23], and 15°C—the threshold at which the probability of eel climbing increases (see also Fig 5A), respectively. Symbols above the figure indicate the moon phase (open and closed circles indicate full moon and new moon, respectively). (B) the number of eels that successfully climbed the weir (closed bars). (C) daily precipitation (open bars).

During our study period, the daily average water temperature ranged from 6.7 to 21.8°C and gradually increased as the season progressed (Fig 2A); mean values of daily average water temperature were 11.2°C, 13.8°C, 16.0°C, and 18.8°C in February, March, April, and May, respectively. In April and May, water temperatures exceeded 13°C—the threshold at which eel behavioral activity decreases [23]—on all days; however, the water temperatures exceeded 13°C for 4 and 24 days in February and March, respectively, whereas in May, the water temperature exceeded 15°C—the threshold at which the probability of eel climbing increases (see the timing of eel climbing in the results section in this study)—on all days; however, the water temperature exceeded 15°C for 1, 6, and 24 days in February, March, and April, respectively. It rained for 2, 11, 7, and 14 days in February, March, April, and May, respectively (Fig 2C).

### Video observations

**Fig 3:**
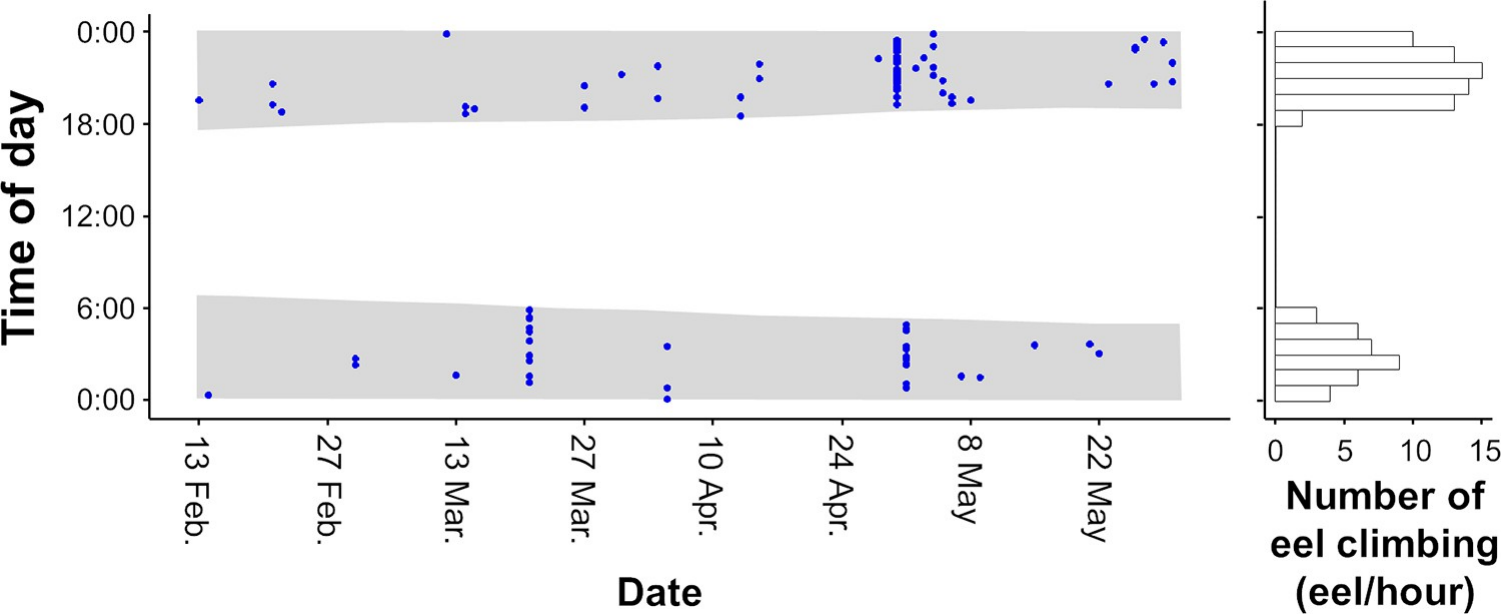
Time plot of the eel climbing occurrence (blue plots) and its hourly frequency (bars). Shaded areas indicate nighttime based on sunrise and sunset times during the study period. Data on sunrise and sunset times were downloaded from the database of the Japan Coast Guard [30].

**Fig 4:**
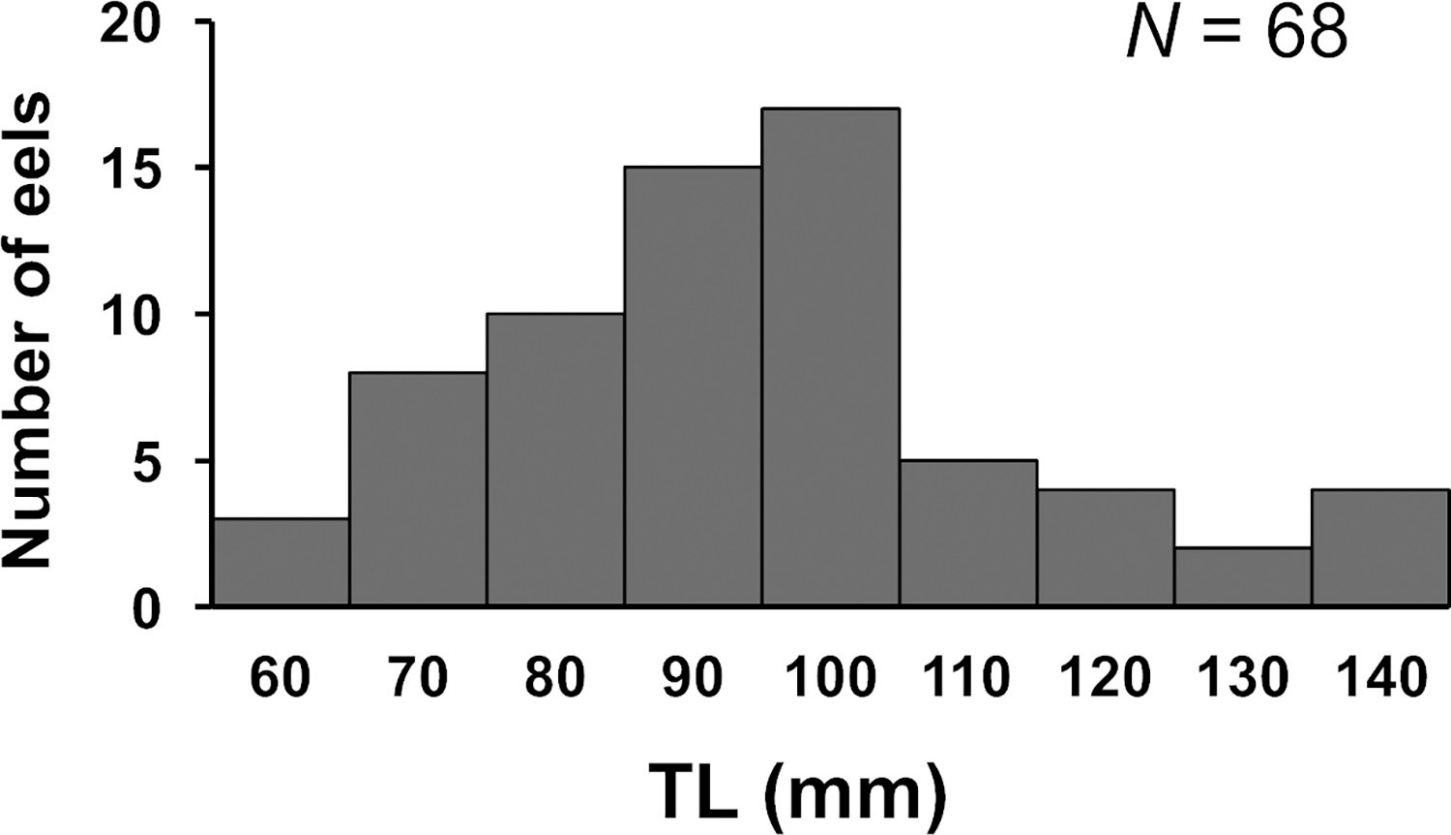
Total length (TL) distributions in eels that successfully climbed the weir.

A total of 102 eels successfully climbed the weir with 165 cm height during our study period, from February 13 to May 30, 2021 (Fig 2B; S2 Data). Within the range of each camera on the left and right bank, all eels were found to climb closer to the edges of the weir (S1 Movie). The number of eels climbing in April (n = 39) and May (n = 38) was higher than that in February (n = 5) and March (n = 20). There were only 3 days in which more than 10 eels climbed in one day throughout our study period: the first one (n = 10; 9.8% of the total number of climbing eels) occurred on March 21, and the second and third ones occurred on April 30 (n = 29; 28.4%) and May 1 (n = 13; 12.7%), 2021. It rained with 31.5 mm/d and 28.5 mm/d precipitation on March 21 and May 1, respectively, and there was a lack of rain on April 30, but precipitation_*t-12h*_ and precipitation_*t-24h*_ were 43.0 mm/day and 92.5 mm/day, respectively (Fig 2C). The video observations clearly indicated that the water levels were higher on these three days. The eels climbed up the edge of the weir, where there was little water flowing, wriggling their bodies. They were also observed resting with their bodies hooked on the protrusions of the wall on their way climbing the weir. All eels climbed the weir at night, between sunset (18:00) and sunrise (6:00) (Fig 3). There were two peaks of climbing eel abundance at night: the first peak (n = 17) occurred after sunset (20:00–21:00), and the second peak (n = 8) occurred before sunrise (3:00–4:00). We were able to measure the TL of 68 eels (out of 102), with a mean TL of 95 mm ranging from 60 to 140 mm (Fig 4).

### Timing of eel climbing

**Fig 5:**
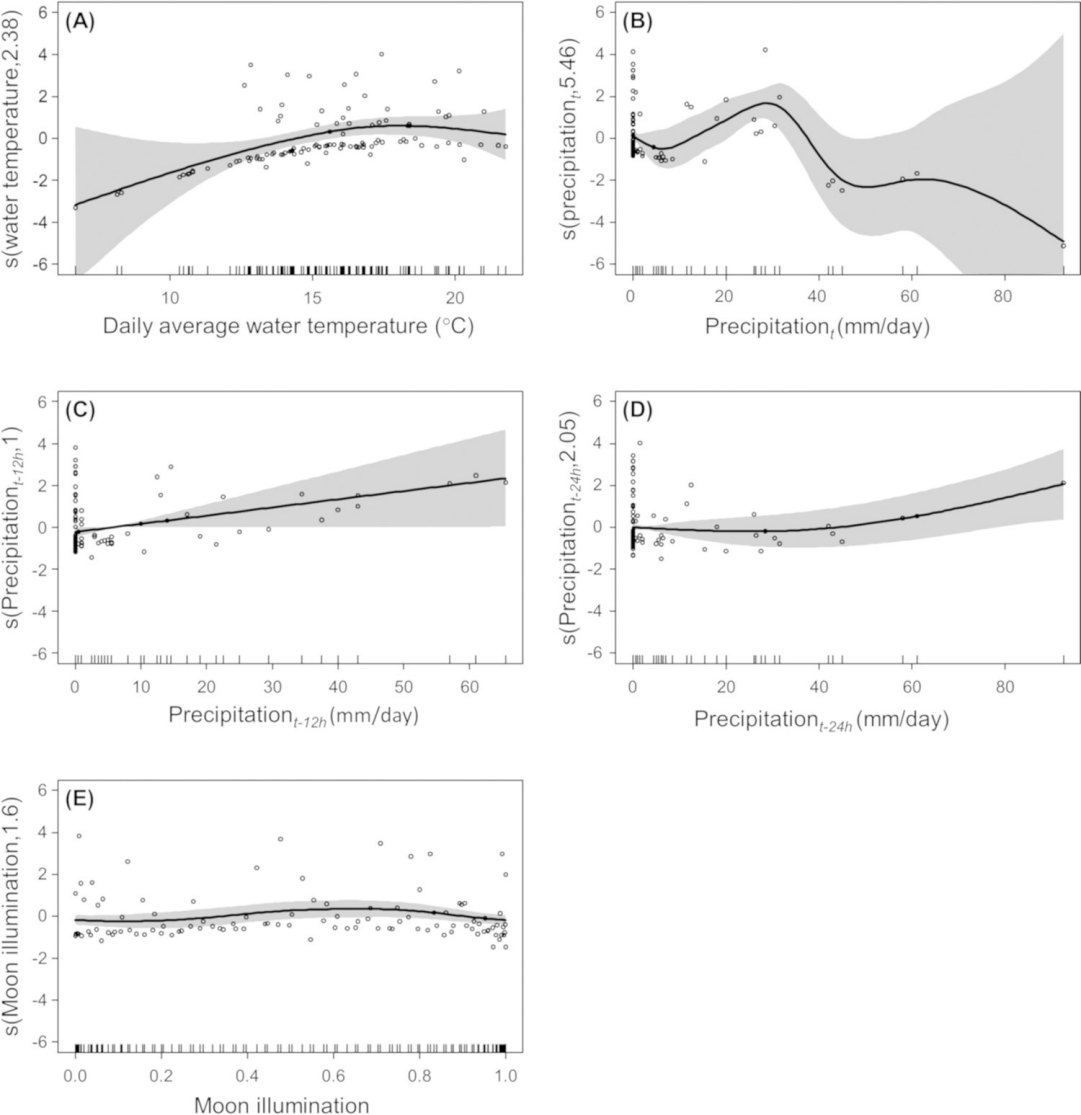
Results of the general additive model on the probability of eel climbing. (A) daily average water temperature, (B) precipitation_*t*_, (C) precipitation_*t-12h*_, (D) precipitation_*t-24h*_, and (E) moon illumination. Circles indicate all observations of eel climbing. Lines with shaded areas indicate estimated curves with 95% confidence bands. Tick marks on the x-axes show the location of observations along the range of continuous explanatory variables.

The GAM results revealed that the water temperature and precipitation were markedly related to the number of eels climbing the weir (Table 1; Fig 5A–5D). The probability of eel climbing increased as the average water temperature increased (*p* < 0.05; Table 1), especially when water temperature exceeded approximately 15°C (Fig 5A). There was a different relation between precipitation_*t*_ and the other precipitation parameters (precipitation_*t-12h*_ and precipitation_*t-24h*_); precipitation_*t*_ had one peak of eel climbing, ranging between ca. 15–40 mm/d (*p* < 0.001; Fig 5B), while the probability of eel climbing increased as both precipitation_*t-12h*_ and precipitation_*t-24h*_ increased (*p* < 0.05 for both; Fig 5C and 5D). However, moon illumination tended to affect the number of eels climbing but with no statistical significance (*p* = 0.052; Table 1; Fig 5E), which implied the weak effect of moon illumination on the eel climbing.

**Table 1:** The effect of environmental variables on eel climbing obtained from the general additive model.

Parametric coefficients	Estimate	Standard error	*t* value	*p* value
(Intercept)	-0.899	0.182	-4.934	<0.001
Approximate significance of smooth terms	edf	Ref.df	*F* value	*p* value
s(average water temperature)	2.383	3.012	2.950	0.037
s(precipitation_*t*_)	5.457	6.422	7.206	<0.001
precipitation_*t-12h*_	1.000	1.000	4.043	0.047
s(precipitation_*t-24h*_)	2.048	2.361	4.318	0.019
s(moon illumination)	1.603	8.000	0.522	0.052

Akaike information criterion = 178.8, adjusted *R*^2^ = 0.878, Deviance explained = 68.7%.

## Discussion

Weirs can prevent the upstream migration of anguillid eels, contributing to the loss of longitudinal connectivity within a watershed depending on the relationship between eel density and weir characteristics along the flow direction [12–16,31]. Previous studies have succeeded in collecting eels at the upper reaches of low-height weirs, suggesting that eels can climb a low-height weir, but the growth stage in which they can do so remains unclear. In this study, we directly observed small-sized (60–140 mm TL) Japanese eels successfully climbing a vertical weir (165 cm height) at night. Furthermore, the timing of eel climbing seemed to be influenced by water temperature and precipitation. Previously, we had observed a high eel density (0.67–1.05 eels/m^2^; TL: 93–641 mm) in the upper reaches of a weir in the Akugawa River [25]. Together, our previous and present findings suggest that eels inhabiting regions upstream of the weir climbed the weir when they were small (60–140 mm TL) and then lived and grew upstream.

The surface of the weir wall in this study was eroded with several protrusions. This surface texture may contribute to the success of eel climbing. In this study, eels were sometimes observed clinging to the rough protrusions of a wall during the process of climbing the weir, possibly to prevent falling. Previous studies have also shown that rough shallow sloping surfaces (e.g., studded plastic, open weave geotextile, and bristle substratum) can help eels climb a weir [17–19,32]. These findings suggest that the roughness of the structural surface is important for eel climbing over natural and artificial barriers. It is also likely that weirs covered with periphyton and plants may help young eels (especially glass eels and elvers) climb weirs [13,14,17]. Matsushige et al. [33] demonstrated that Japanese eels were likely to climb the moss-covered rock wall of a natural waterfall (46 m in height) because eels were collected from an upper reach of the waterfall where they were not stocked. Thus, the surface structure of the weir wall is pertinent for the success of eel climbing.

In this study, Japanese eels with over 150 mm TL were not observed climbing vertical weirs. One hypothesis is that eels have a TL threshold for climbing a weir. Eels over 150 mm TL may fall while trying to climb a weir because they cannot support their own weight. This hypothesis is supported by the experimental study of Lagarde et al. [19], who showed that the probability of climbing experimental slopes decreased for marbled eels *Anguilla marmorata* with more than 300 mm TL. Another hypothesis is that eels over 150 mm TL did not try to climb a weir. Small-sized eels (glass eels, elvers, and small yellow eels) are in the process of being dispersed over a wide range of habitats, from coastal areas to upstream river basins after they, as glass eels, reach coastal areas [9]. On the other hand, large eels have strong site fidelity and a relatively small home range [23], similar to other anguillid eels [34,35]. In the case of the European eel *Anguilla anguilla*, small-sized eels (less than 240 mm TL) were observed to migrate upstream, but large-sized eels more than 240 mm TL were not [36]. The body size at which eels begin to exhibit a sedentary lifestyle is likely to vary across rivers [36,37]. If eels inhabiting the Akugawa River begin to show sedentary behavior when they reach TL over 150 mm, the second hypothesis is more likely to be the case. However, we could not test these hypotheses in the present study as further studies are required to confirm them.

In this study, all eels climbed a weir at night, with two peaks just after sunset and before sunrise. Similarly, in previous studies, the behavioral activity of eels increased at night, with two peaks just after sunset and before sunrise in Japanese eels as well as other eel species [23,34,35]. Noda et al. [24] reported the diel movement of yellow Japanese eels; eels move to a river after sunset and return to a brackish lagoon just before sunrise, possibly for foraging purposes [38]. However, the two peaks of eel climbing are unlikely to be explained only by foraging behavior because eel climbing is likely to be an energy- and time-consuming activity. One possible reason for the occurrence of eel climbing at night is to minimize predation risk [39]. In a previous study, Japanese eels seemed to reduce predation risk by potential diurnal predators, such as birds (the grey heron (*Ardea cinere*) and kingfisher *(Alcedo atthis)*) [40,41] and fish (the largemouth bass *Micropterus salmoides*) [42], when they climbed a weir.

Previous studies have revealed that the upstream migratory behavior of anguillid eels appears to be strongly related to water temperature and that the threshold varies across eel species [31,43–46]. In European eels, the upstream locomotor activity at 18°C was higher than that at 10°C [47]. In yellow Japanese eels over 500 mm TL, behavioral activity decreases below approximately 13°C [23]. The threshold water temperature for wall climbing of glass eel, *Anguilla* spp., from Iceland was between 12°C and 14.5°C [44]. In this study, frequent eel climbing was observed from March to May when water temperature exceeded approximately 13°C, and our GAM results revealed that the probability of eel climbing increased when water temperature exceeded approximately 15°C. Therefore, this study confirms that an increase in water temperature is a key environmental factor in activating upstream migration, including climbing behavior in eels.

Precipitation is an important factor that stimulates upstream migration in several fish species, including anguillid eels [48]. Our GAM results also revealed the positive effects of precipitation on the probability of eel climbing. It was thought that too much rainfall would impede eel climbing because the river discharge would be too strong [43,45,46,49]. A possible explanation for eels climbing the weir during increased river discharge is to avoid predation risk. When it rains, the water level rises, and the river water becomes turbid. In such situations, both bird and fish predators cannot detect aquatic prey, thereby decreasing their feeding rate [41,50]. In this study, we could not use the flow rate as an explanatory variable in GAM. Further study is needed in this area.

Our GAM results revealed that the lunar illumination was ineffectual regarding the probability of eel climbing. Previous studies reported that American eels exhibited high activity of upstream movement under low lunar illumination, that is, a new moon [46,49]. These previous and present studies used cloud cover, lunar phase, and/or lunar illumination as alternative indicators of illumination in a water column [46,49]. The alternative indices of illumination used in these studies may have been insufficient to explain the factors stimulating the upstream migration of eels because illumination in the water column is possibly affected by turbidity in the water column, water level/discharge, weather (i.e., cloudy), moon phase, moon illumination, and its interactions. Therefore, further studies on the relationship between the ambient light environment and eel upstream migration, including eel climbing, are required.

In the present study, all eels were found to climb through the edges of the weir when they were wet during or after rainfall. However, this study had two limitations regarding the visibility of the recorded video. First, it was difficult to confirm the eel climbing through the weir during rainy days because of the surge in water level and water velocity. A previous study revealed that the burst swimming speed of Japanese eel elvers ranged from 36–56 cm/sec [51], which can potentially be slower than the increased river flow after rainy days. In fact, the average current velocity at the center of the river just above the weir measured on September 5, 2022, three days after the rainfall (total 53.0 mm on 30 August 30 to September 2, 2022), was 43.8 cm/sec (range: 43.2–44.4 cm/sec). Therefore, the initial assumption was that the eels could not climb the center of the weir on rainy days since the current speed at the center of the weir exceeded the burst swimming speed of Japanese eels. Our results that eels climbed to areas with less water (less easily washed away) closer to the edges even when water levels were low was supported to this assumption. In addition, the camera’s shooting range could not capture the center of the weir. Thus, we could not discount the possibility that eels climbed the center of the weir on sunny days.

Video imaging is an effective tool for wild animal research, including studies on animal diversity, abundance, and behavior [52]. Being non-invasive, video imaging allows for target species to be studied in their habitats without any disturbance [53,54]. Recently, ecological monitoring using video systems has become more applicable in a broader range of situations owing to the technological developments in cameras, such as extended battery life and increased storage capacity [55,56]. This method has been used in ecological studies of anguillid eels [57,58] and other fish species [59]. However, this monitoring technique has certain disadvantages. It takes a lot of time and effort to manually obtain data from recorded videos [58]. In this study, it took approximately 200 h to check all videos from two cameras that were continuously recording for four months. Provided that better technologies are applied for the automatic or semi-automatic detection of target species (Japanese eels) in recorded videos (i.e., machine learning techniques and models), video surveys will be extensively used in field ecology studies.

Overall, in this study, we recorded Japanese eels climbing a low-height vertical weir using infrared video camera systems. The infrared video camera system used in this study can be used for monitoring activities at the numerous weirs in rivers across Japan. In this study, small-sized Japanese eels ranging from 60 to 140 mm TL could climb a weir (165 cm height) at night. It should be noted that a weir can still act as a physical barrier to the upstream movement of small-sized eels. Some small-sized eels can climb a weir while others cannot climb or stay downstream of a weir, resulting in a reduction in eel density with increasing the number of weirs or their cumulative heights, as shown by previous studies [12–15]. Moreover, the water temperature and precipitation seemed to be important for stimulating the eel’s climbing behavior. However, we used only a limited number of environmental factors for GAM analysis; thus, other abiotic (e.g., illuminance in the water column, tidal phase, and atmospheric pressure) and biotic (e.g., eel and/or predator density, and food and/or shelter availability) factors that we did not use in this study may be complexly related to eel climbing and upstream migration of anguillid eels. Thus, further studies on abiotic and biotic factors that stimulate eel climbing are required. Furthermore, this study could not confirm the success rate of eel climbing and the threshold of weir height that eels could climb. Therefore, further studies on the effects of weirs on eel upstream movement and distribution within a river would help enhance the knowledge required for the comprehensive management and conservation of wild eel stocks.

## Supporting information

S1 Data (XLSX)

S2 Data (XLSX)

S1 Movie Video of the eel that climbed successfully (30 April 2021). This video was played at an 8x speed. The arrow indicates an eel that climbed successfully.
(MP4)
